# Sympathetic Nervous System Catecholamines and Neuropeptide Y Neurotransmitters Are Upregulated in Human NAFLD and Modulate the Fibrogenic Function of Hepatic Stellate Cells

**DOI:** 10.1371/journal.pone.0072928

**Published:** 2013-09-03

**Authors:** Barbara Sigala, Chad McKee, Junpei Soeda, Valerio Pazienza, Maelle Morgan, Ching-I Lin, Clare Selden, Sara Vander Borght, Gianluigi Mazzoccoli, Tania Roskams, Manlio Vinciguerra, Jude A. Oben

**Affiliations:** 1 Institute for Liver and Digestive Health, University College London, Royal Free Hospital, London, United Kingdom; 2 Gastroenterology Unit, IRCCS “Casa Sollievo della Sofferenza”, Hospital San Giovanni Rotondo (FG), San Giovanni, Italy; 3 Department of Pathology, Laboratory of Morphology and Molecular Pathology, University Hospitals of Leuven, Leuven, Belgium; 4 Department of Medical Sciences, Division of Internal Medicine IRCCS Scientific Institute and Regional General Hospital “Casa Sollievo della Sofferenza”, San Giovanni Rotondo (FG), Italy; 5 Department of Gastroenterology and Hepatology, Guy's and St Thomas' Hospital, London, United Kingdom; University of Verona, Ospedale Civile Maggiore, Italy

## Abstract

**Background:**

*S*ympathetic nervous system (SNS) signalling regulates murine hepatic fibrogenesis through effects on hepatic stellate cells (HSC), and obesity-related hypertension with SNS activation accelerates progression of non-alcoholic fatty liver disease (NAFLD), the commonest cause of chronic liver disease. NAFLD may lead to cirrhosis. The effects of the SNS neurotransmitters norepinephrine (NE), epinephrine (EPI) and neuropeptide Y (NPY) on human primary HSC (hHSC) function and in NAFLD pathogenesis are poorly understood.

**Aims:**

to determine the mechanistic effects of NE/EPI/NPY on phenotypic changes in cultured hHSC, and to study SNS signalling in human NAFLD livers.

**Methods:**

Freshly isolated hHSC were assessed for expression of cathecholamine/neuropeptide Y receptors and for the synthesis of NE/EPI. The effects of NE/EPI/NPY and adrenoceptor antagonists prazosin (PRZ)/propranolol (PRL) on hHSC fibrogenic functions and the involved kinases and interleukin pathways were examined. Human livers with proven NAFLD were then assessed for upregulation of SNS signalling components.

**Results:**

Activated hHSC express functional α/β-adrenoceptors and NPY receptors, which are upregulated in the livers of patients with cirrhotic NAFLD. hHSC in culture synthesize and release NE/EPI, required for their optimal basal growth and survival. Exogenous NE/EPI and NPY dose-dependently induced hHSC proliferation, mediated via p38 MAP, PI3K and MEK signalling. NE and EPI but not NPY increased expression of collagen-1α2 via TGF-β without involvement of the pro-fibrogenic cytokines leptin, IL-4 and IL-13 or the anti-fibrotic cytokine IL-10.

**Conclusions:**

hHSC synthesize and require cathecholamines for optimal survival and fibrogenic functionality. Activated hHSC express directly fibrogenic α/β-adrenoceptors and NPY receptors, upregulated in human cirrhotic NAFLD. Adrenoceptor and NPY antagonists may be novel anti-fibrotic agents in human NAFLD.

## Introduction

The sympathetic nervous system (SNS), a major branch of the autonomic nervous system, is responsible for up- and down-regulating many homeostatic mechanisms in living organisms. Pre- and post-ganglionic neurons are involved in the transmission of signals through the sympathetic system. At the synapses within the ganglia, preganglionic neurons release acetylcholine, a neurotransmitter that activates nicotinic acetylcholine receptors on postganglionic neurons. In response to this stimulus postganglionic neurons release catecholamines such as norepinephrine, which activate adrenergic receptors on the target tissues. The activation of target tissue receptors causes the effects associated with the sympathetic system such as vasoconstriction and hypertension. Hepatic stellate cells (HSC), also known as peri-sinusoidal cells or Ito cells, are pericytes found in the peri-sinusoidal space between the sinusoids and hepatocytes in the liver. HSC are the principal hepatic fibrogenic cells generating scar tissue in response to persisting liver injury [1]. Emerging evidence suggests that HSC express functional adrenoceptors [2], [3], and SNS neurotransmitters induce *in vitro* HSC characteristic pro-fibrotic phenotypic changes [4], [5]. Additionally, mice with genetic deletion of dopamine-β-hydroxylase, lacking cathecholamines, are poorly fibrogenic [2]. Neuropeptide Y (NPY), co-released with cathecholamines at SNS nerve terminals modulates in turn cathecholamine release [6], [7] and induces murine HSC proliferation but not collagen expression [5]. The applicability of these findings to human liver fibrosis is unknown. Nearly 4 decades ago it was envisaged that SNS activation is an adaptive response to overeating helping to stabilise body weight [8] with underfed rodents suppressing their SNS activity. Similar effects observed in humans provide support for a key role for the SNS in obesity. Overfeeding concurrently stimulates cardiac sympathetic nerves, which are proposed to be a reflex response to circulatory overload [9], [10]. Hypertension observed with SNS activation is a component of the metabolic syndrome and obesity [9]–[11]. In 90% of cases obesity is associated with non-alcoholic fatty liver disease (NAFLD), an accumulation of intra-hepatic triglycerides that is often considered the hepatic manifestation of insulin resistance. NAFLD is the most common cause of chronic liver disease in Western countries with up to one third of the US population affected [12]. NAFLD is a spectrum of hepatic disorders that encompass varying degrees of liver damage ranging from steatosis to non-alcoholic steatohepatitis (NASH) characterized by hepatocellular injury and fibrosis which may progress to cirrhosis and hepatocellular carcinoma [13]–[15]. There is currently no pharmacological treatment for NAFLD, other than loss weight and physical exercise [16]. The aim of this study was to determine if human HSC (hHSC) synthesize and respond to cathecholamines or NPY, and explore the signalling pathways therein involved. Moreover, we have studied changes in SNS signalling components in liver specimens from NAFLD patients.

## Materials and Methods

### Isolation and culture of hHSCs

Human HSCs were isolated as described [17] from resected normal liver tissue, in patients undergoing clinically indicated resection of liver metastases. These liver specimens were obtained with appropriate local Ethics Committee approval (UCL – Royal Free Hospital, London, UK). Written informed consent from the donors was obtained for use of samples in research. Experiments were performed with hHSCs in culture at 7–30 days. Proliferation experiments were performed at first hHSC activation [5], [18] and not on multiply passaged cells. A subset of experiments was performed with freshly isolated, quiescent HSCs.

### Near normal and cirrhotic non-alcoholic steatohepatitis liver specimens

With appropriate ethical approval and consent for research, liver specimens were obtained from adult patients undergoing clinically indicated investigations for NAFLD or liver transplantation in our units. A total of 10 human liver specimens were used in the current study: 5 were from liver biopsies in patients in whom the final diagnosis was near normal liver – Brunt-Kleiner [19] NAFLD liver fibrosis score  = F0; and 5 were from peri-transplant resected specimens in whom the final diagnosis was cirrhotic NAFLD, BK NAFLD fibrosis score  = F4. The histopathological evaluations were performed by an expert liver histopathologist.

### Immunocytochemistry and confirmation of hHSC identity

hHSC were prepared and stained for alpha smooth muscle actin (ASMA) and glial fibrillary acidic protein (GFAP) with controls as described [5], [18]. Images were captured with a Zeiss Axiophot microscope and a Leica DMRE fluorescence microscope fitted with an SP confocal head.

### High performance liquid chromatography (HPLC) analysis

Cathecholamines were extracted from HSC conditioned medium as described [2] and an aliquot was injected into a reverse phase ion pair HPLC system with electrochemical detection using ESA Coulochem5100 A.

### Cell proliferation assay

Quantitative hHSC proliferation assays were performed with the WST-8 cell counting kit (Dojindo Molecular Technologies, NBS Biologicals, Huntingdon, Cambridgeshire, UK) as described [5], [18].

### Apoptosis assay

hHSC (5×10^5^/ ml) were plated into 6 mm petri dishes with test agents. Cells without serum served as positive control of apoptosis and cells cultured with PDGF served as a negative control of apoptosis. At harvest, 48 hours later, apoptotic activity was assessed with the Vybrant (annexin V) apoptosis assay kit 2 (Molecular Probes, Invitrogen). FACS analysis was performed using a Becton-Coultor flow cytometer.

### RT-PCR

RT-PCR was performed as previously described [20]. RNA was isolated from activated hHSCs using TRizol (Invitrogen) and cDNA synthesized using QuantiTect Rev. Transcription kit (Invitrogen). Quantitative RtPCR was performed using a Rotorgene RG-3000 instrument (Corbett Research), SYBR GreenER and a 2 Step qRT-PCR kit (Invitrogen). Target gene levels in treated samples are presented as a ratio to levels detected in corresponding control samples, according to the ΔΔCt method. For semi-quantitative PCR, Superscript III one-step RT-PCR with platinum Taq kit (Invitrogen) and classic II 18s internal standard kit (Ambion) was used. The cycle number and 18 S primer/ competimer pair ratio for each primer set were determined by the manufacture's instruction. PCR products were separated by electrophoresis and quantified by densitometry using LabWorks 4.6 software (UVP, USA). Primer sequences, annealing temperatures and products sizes are as shown in Tables 1 and 2.

**Table 1:** RT-PCR primers used to amplify adrenoceptors transcripts.

Gene	Primer sequences (5′3′)	Annealing (°C)	Product size (bp)
**Collagen 1α2**	Sense: GAA CGG TCC ACG ATT GCA TG Antisense: GGC ATG TTG CTA GGC ACG AAG	55	167
**TGF-β1**	Sense: CTA CTA CGC CAA GGA GGT CAC Antisense: TTG CTG AGG TAT CGC CAG GAA	55	246
**α1A**	Sense: TGG CCG ACC TCC TGC TCA CCT C Antisense: GGC CCC GGC TCT CCC TCT TG	55	444
**α1B**	Sense: CCC CCG ACG CCG TGT TCA AGG TG Antisense: CTC AGG CGC GGG CAG GCT CAG GA	55	407
**α1D**	Sense: AGC GCT TCT GCG GTA TCA Antisense: GGA GGA AGG CGC GCT TGA AC	55	516
**β1**	Sense: GTG GCC CTG CGC GAG CAG AA Antisense: GCG GCA GTA GAT GAT GGG GTT GA	55	195
**β2**	Sense: TCA TCA CTT CAC TGG CCT GT Antisense: CTT GGT CAG CAG GCT CTG GT	55	230
**β3**	Sense: GTC GTT TGC GCC CAT CAT GA Antisense: AGC AGA GAG TGA AGG TGC CC	55	410
**IL-4**	Sense: GAA GAG AGG TGC TGA TTG GC Antisense: GGT TCC TGT CGA GCC GTT TC	53	530
**IL-10**	Sense: CAT CAA GGC GCA TGT GAA CT anti-sense: CTT GGA ATG GAA GCT TCT GT	53	590
**IL-13**	Sense: GAG ACA GGA CCT GAC TAT TG anti-sense: AGA ATT CTG TAC ACA GTA CT	53	540
**GAPDH**	Sense: AGT ATG ACT CCA CTC ACG GCA A Antisense: TCT CGC TCC TGG AAG ATG GT	55	100

**Table 2:** RT-PCR primers used to amplify neuropeptide Y receptors (NPY) transcripts.

Gene	Primer sequences (5′3′)	Annealing (°C)	Product size (bp)
**NPY1R**	Sense: GAAAATCATTCAGTCCACTC Antisense: GGATGTTGGTAACATTTCTC	55	205
**NPY2R**	Sense: GAGTATTCGCTGATTGAGAT Antisense: GGTTCTTCAATTTACTCCAA	55	184
**NPY4R**	Sense: CTTCCTACAGCATTGAGACT Antisense: AAAGATCCAGTAGTCCATGA	55	191
**NPY5R**	Sense: ACTACGGTAAACTTCCTCA Antisense: TGACACACATTGAAGAAAAG	55	154
**NPY6R**	Sense: CCACATGTATAAACCCTCTC Antisense: GTTGTTATACGAGCCAATCT	55	175
**GAPDH**	Sense: AGT ATG ACT CCA CTC ACG GCA A Antisense: TCT CGC TCC TGG AAG ATG GT	55	100

### Western blotting

Western blotting analyses were performed as previously described [21]. Aliquots of hHSC were homogenized with Radio Immunoprecipitation Assay (RIPA) buffer (150 mM NaCl, 20 mM Tris-HCL, 1% sodium deoxycholate, 0.1% SDS, and 1% NP-40, Sigma, UK) containing protease inhibitors (Roche Applied Science, UK). Samples (30 μg protein) were prepared according to the manufacturer's instructions and loaded onto precast 10% bis-tris polyacrylamide gels (Invitrogen, UK). Western-blotting was performed with NuPage Electrophoresis System (Invitrogen, UK) and blots incubated with rabbit primary anti-β1 and anti-β2 adrenergic receptor antibodies (1×600 and 1×400, respectively; Santa Cruz Biotechnology, USA), goat anti-β3 adrenergic receptor antibody (1×400; ProSci, USA), rabbit anti-NPY1 receptor antibody (1×500; Abcam, UK), goat anti-NPY4 and anti-NPY6 receptor antibodies (1×500, Santa Cruz Biotechnology, USA), and rabbit anti-β-actin antibody (1×1000; Cell Signaling Technology, UK). Horseradish peroxidase-conjugated secondary antibodies, goat anti-rabbit IgG (Santa Cruz Biotechnology, USA) and bovine anti-goat IgG (Jackson ImmunoResearch Laboratories, USA) were used to detect primary antibodies. Signals were detected using ECL Western Blotting Detection Reagents (GE Healthcare, UK). Equal loading (30 μg) was verified by comparing expression levels of β-actin.

### Immunohistochemistry

Immunohistochemistry staining was performed as previously described [22], [23]. Briefly, four-µm-thick frozen sections of human liver were cut, dried overnight at room temperature, fixed in acetone and washed in PBS. All sections were incubated, 30 minutes, room temperature with primary antibodies. Primary antibodies (Table 3) were from Santa Cruz Biotech, Heidelberg, Germany. For DBH, β1- β2 and β3-AR samples were incubated with an anti–rabbit peroxidase–conjugated EnVision antibody (Dako) 30 minutes/room temperature. For α1A-AR, α1B-AR, α1D-AR and β3-AR, we used a three-step immunoperoxidase procedure: peroxidase labeled rabbit-anti goat IgG (Dako, Denmark) followed by peroxidase labeled swine anti-rabbit IgG (Dako). The secondary and tertiary antibodies were diluted (1×50 and 1×100 respectively) in PBS, pH 7.2 containing 10% normal human serum. For negative controls the primary antibody was omitted. Number of hHSC positive for protein expression of α1A, β1, β2 and β3 adrenoceptors in livers of patients with NASH compared to near normal controls were assessed as previously described [24].

**Table 3:** List of antibodies, and relative dilutions, utilized for immunoblotting.

Antibody	SCBT Catalogue No	Source	Primary Antibody Dilution
α_1A_-AR (C-19)	SC-1477	Goat polyclonal	1:20
β1-AR (V-19)	SC-568	Rabbit polyclonal	1:200
β2-AR (H-73)	SC-9042	Rabbit polyclonal	1:100
β3-AR (C-20)	SC-1472	Goat polyclonal	1:10
DβH (H-213)	SC-15317	Rabbit polyclonal	1:100

### Induction of leptin, IL-4, IL-10, IL-13 gene expression and induction of leptin or IL-13 protein expression by NE

hHSC were cultured under conditions identical to those used above to detect NE effects on hHSC proliferation. After 48 hours, RNA was extracted from the cells and analysed by Q-RT-PCR for expression of leptin, IL4, 10 and 13. Additionally, NE conditioned hHSC media was assayed by ELISA for leptin and IL-13 with a methodology as supplied – R&D systems, Abingdon, UK.

### Drugs

All from Sigma, unless stated otherwise.

### Statistical analysis

Data expressed as mean (±SEM). Statistical analyses performed using the Mann-Whitney test, significance accepted as p<0.05.

## Results

### Characteristics of cultured hHSC

**Figure 1:**
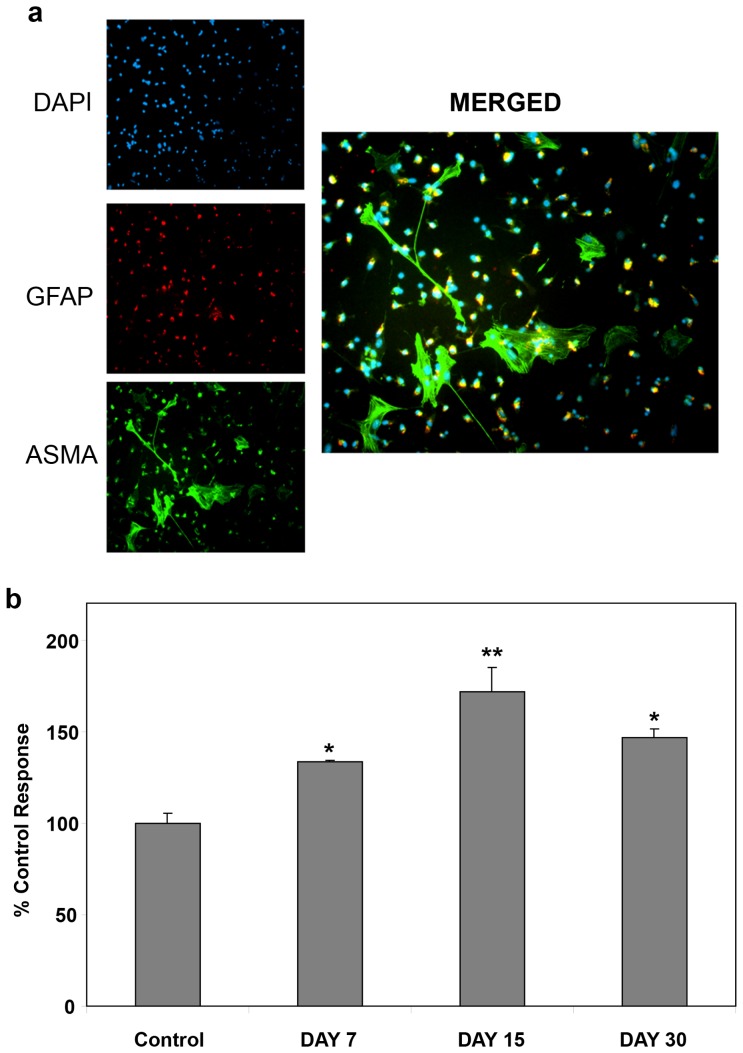
Characteristics of cultured hHSC: hHSC express GFAP plus ASMA and basal proliferative activity of hHSC is maximal at after 15 days in culture and reduces thereafter. **a**) Freshly isolated cells hHSC in culture were confirmed as HSC by auto-fluorescence, and expression of ASMA and GFAP verified at day 4, by immunocytochemistry. **b**) Basal proliferative activity of hHSC was maximal at day 15 (non-passaged, not fully activated), compared to day 7 (non-passaged, not fully-activated) or day 30 (passaged, fully-activated) with basal proliferative activity less at day 30 compared to day 15. *p<0.05, **p<0.0001, n = 5, compared to control response.

Upon isolation, primary hHSC cell identity and activation was confirmed by their expression of established indicators, ASMA and GFAP, verified at day 4 by immunocytochemistry (Figure 1a). We confirmed with quantitative RT-PCR that after 10 days in culture these cells had increased expression of ASMA by about 600%, decreased GFAP expression by about 50% and increased collagen gene expression by about 400% compared to freshly isolated cells at day 0 (results not shown). We then studied the basal proliferative activity of hHSC after 7, 15 or 30 days in culture (Figure 1b). Basal hHSC proliferation was maximal at day 15, compared to day 7 or 30 (Figure 1b). Therefore, subsequent proliferation experiments were performed with hHSCs at day 15.

### Primary hHSC express adrenoceptors and NPY receptors which are upregulated in NAFLD Cirrhosis

**Figure 2:**
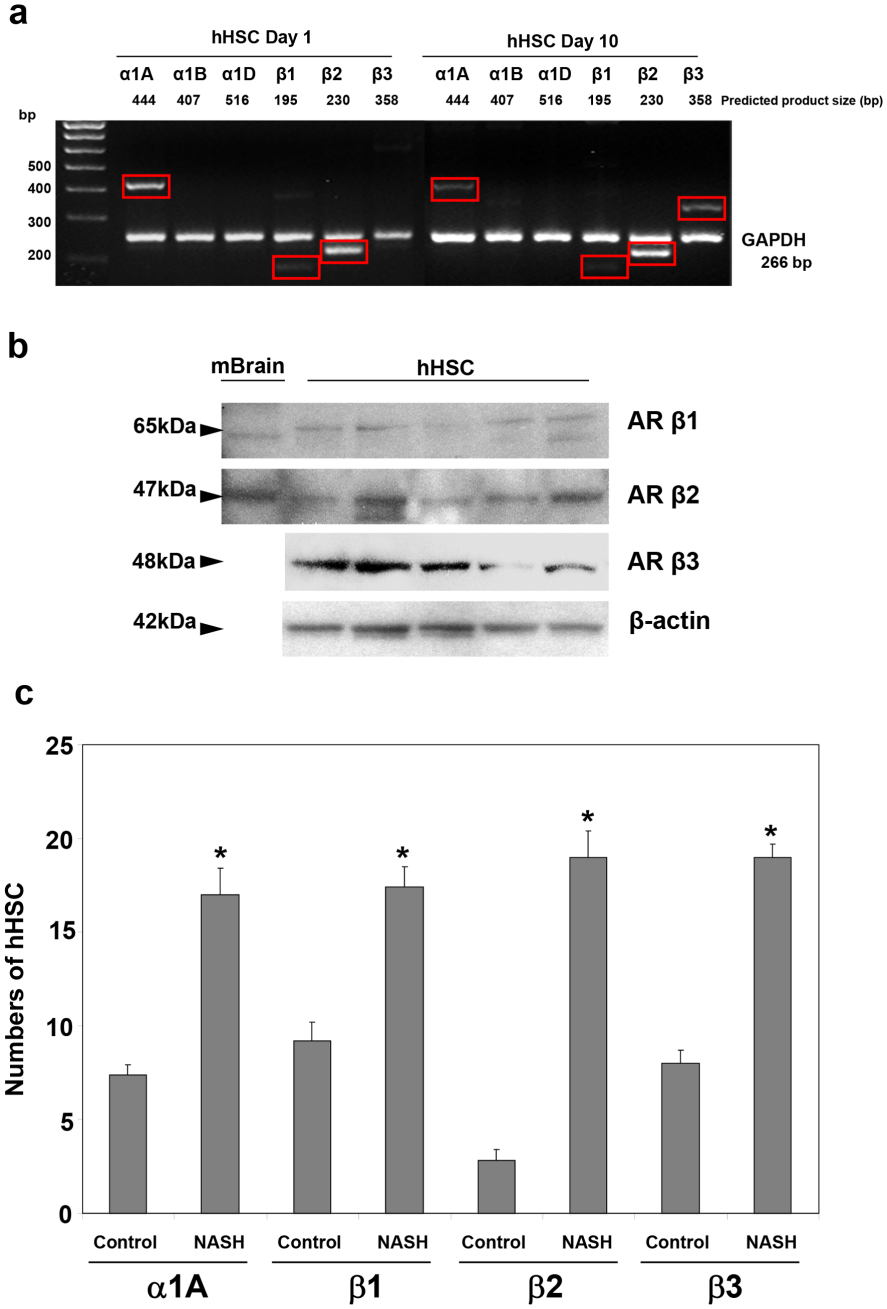
Primary hHSC express adrenoceptors and NPY receptors, upregulated in NAFLD cirrhosis. **a, b**) Semi-quantitative Rt-PCR and Western blot analyses showing that cultured activated hHSC express α1A, β1, β2 and β3 adrenoceptorsm but not absent α1B or α1D adrenoceptors as confirmed by protein level expression. PCR reactions were carried out for 35 cycles. **c**) Immunohistochemistry for hHSC, showed significant upregulation of α1A, β1, β2 and β3 in livers of patients with NASH cirrhosis patients compared to near normal controls. *p<0.05.

We previously showed that murine HSC express mediocre amounts of α1A, but exuberant α1B, α1D, β1 and β2 adrenoceptor subtypes [2]. The presence of some adrenoceptor subtypes was recently shown in both activated hHSC and human whole liver [3]. Here, we found by semi-quantitative RT-PCR that culture activated hHSC express α1A, but had no detectable expression of α1B or α1D adrenoceptors (Figure 2a). We also observed expression of β1 and β2 adrenoceptors (Figure 2a). To confirm protein level expression of these receptors, and assay their possible functional significance *in vivo*, we first performed Western blot analysis on hHSC lysate, and then immunohistochemistry for hHSC on clinical liver specimens, either with a nearly normal histology – fibrosis score, F0, or having NAFLD cirrhosis – fibrosis score, F4. Western blot analysis confirmed protein level expression of the β-adrenoceptors (Figure 2b) and immunohistochemistry similarly showed a significantly upregulated number of hHSC positive for protein expression of α1A, β1, β2 and β3 adrenoceptors in livers of patients with NAFLD cirrhosis compared to near normal controls (Figure 2c).

**Figure 3:**
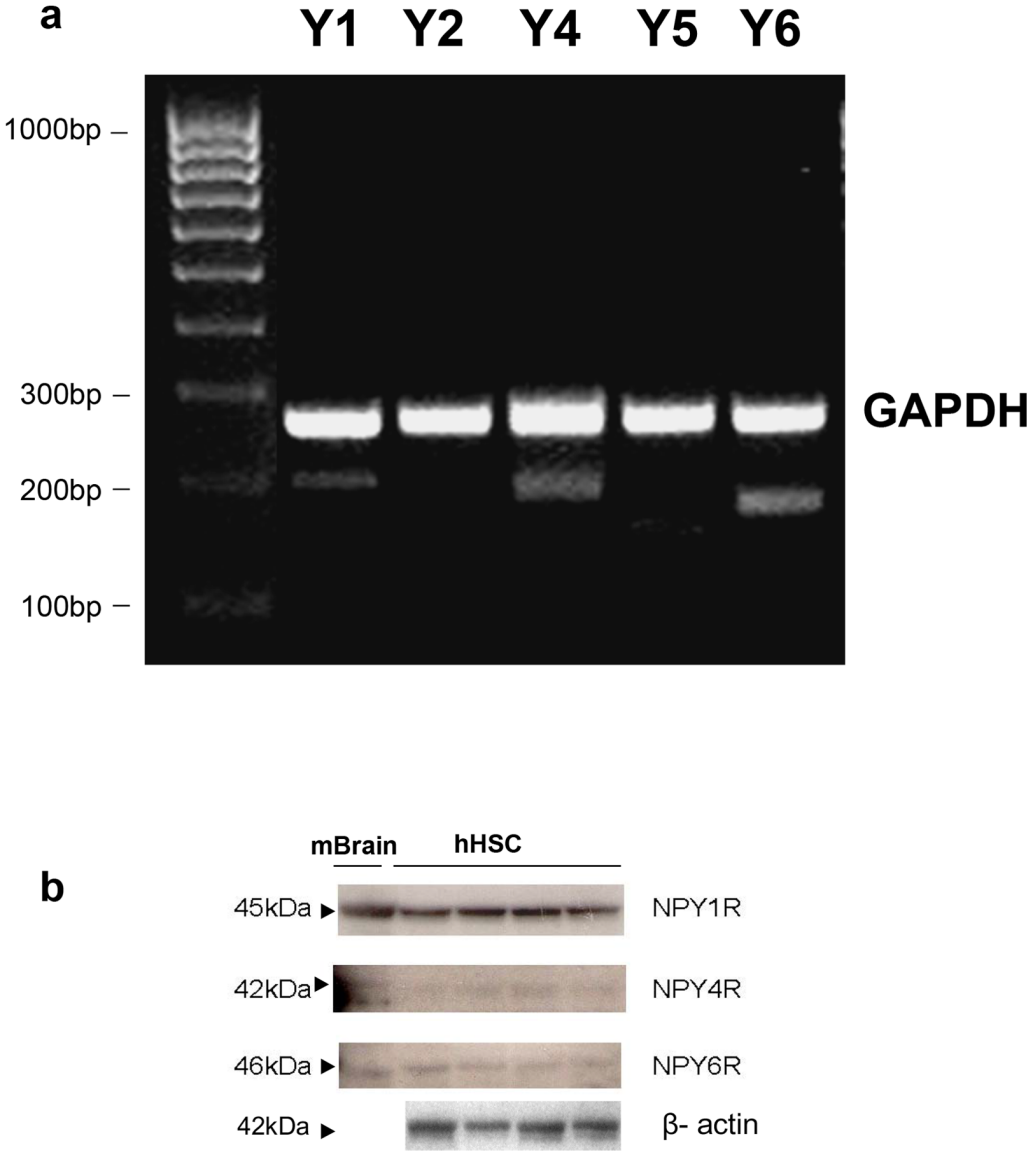
Expression of NPY receptors in hHSC. **a, b**) hHSC also express NPY receptors with abundant Y1, Y4, and Y6 NPY receptors at mRNA and protein levels with little or absent expression of Y2 and Y5.

**Figure 4:**
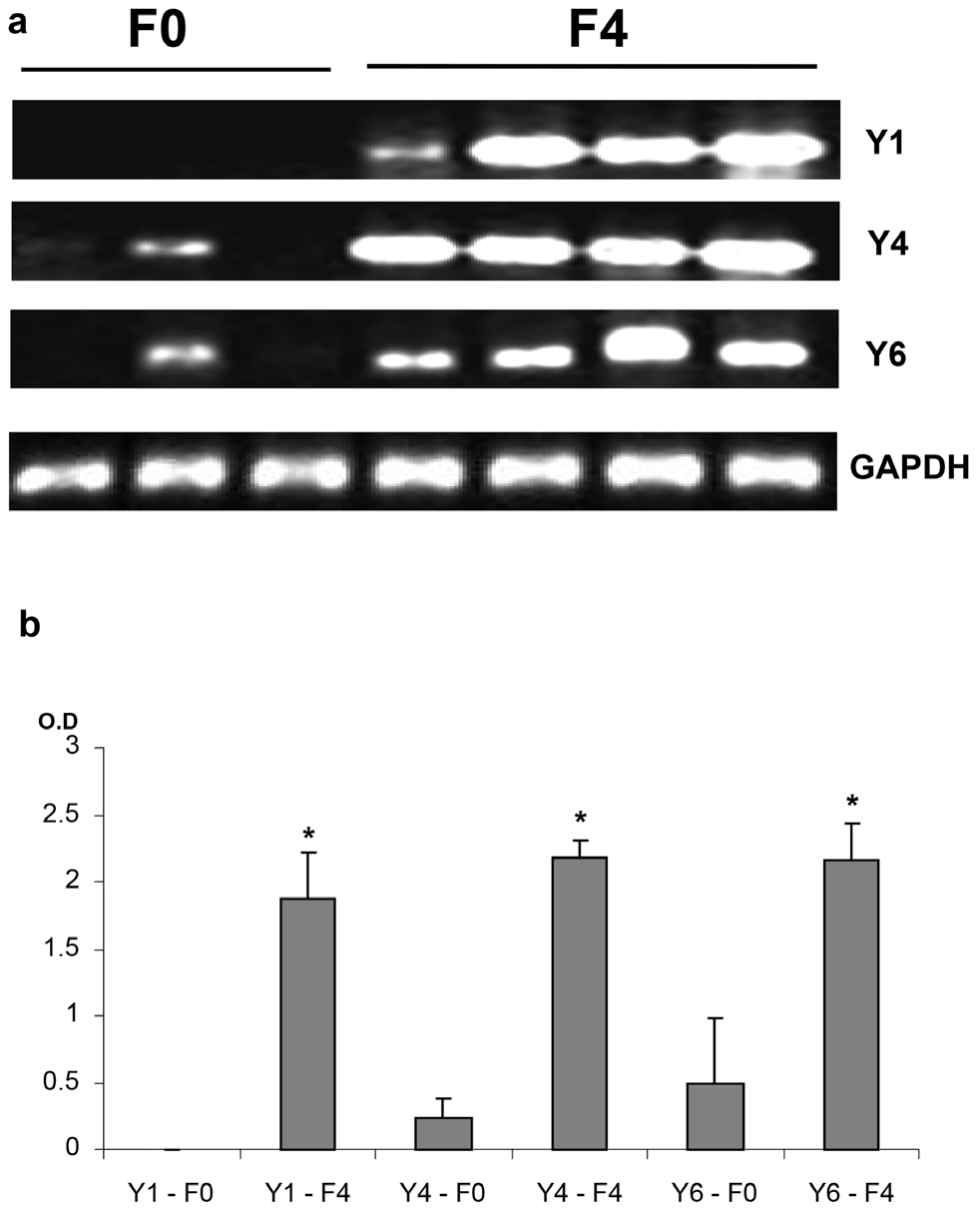
Primary hHSC expressed NPY receptors are upregulated in NAFLD. Semi-quantitative RT-PCR analysis, each lane normalised against its corresponding GAPDH, of liver RNA from patients with NASH fibrosis scored as F0 (n = 3 patients) or F4 (cirrhosis, n = 4 patients) showing human livers with F0 fibrosis having little expression of Y1, Y4 or Y6 NPY but exuberant expression of Y1, Y4 and Y6 NPY in F4 fibrosis (cirrhosis); *p<0.001 compared to F0 control for each receptor subtype, O.D.  =  optical density.

We have previously shown that NPY, a SNS neurotransmitter co-released with NE [7], induces murine HSC proliferation. The expression of NPY receptors by hHSC is not known. Here, we found that hHSC robustly express Y1, Y4, and Y6 NPY receptors with little or no expression of Y2 and Y5 (Figure 3a, b). To confirm the functional relevance of these receptors, we now analysed liver RNA extracted from liver biopsies in patients with near-normal livers, fibrosis score F0 (n = 3 patients) or cirrhotic NAFLD with fibrosis score  = F4 (n = 4 patients). As shown in Figure 4a and b, patients with F0 fibrosis had little expression of Y1, Y4 or Y6 NPY receptors whereas patients with F4 fibrosis (cirrhosis) had exuberant expression of Y1, Y4 and Y6 NPY receptors indicating that some NPY receptors as with adrenoceptors may be implicated in the pathogenesis of NAFLD fibrosis since these receptors are upregulated with fibrosis stage.

### Primary hHSC express the cathecholamine synthesising enzyme dopamine-β-hydroxylase and synthesize/release NE plus EPI to regulate hHSC basal growth

**Figure 5:**
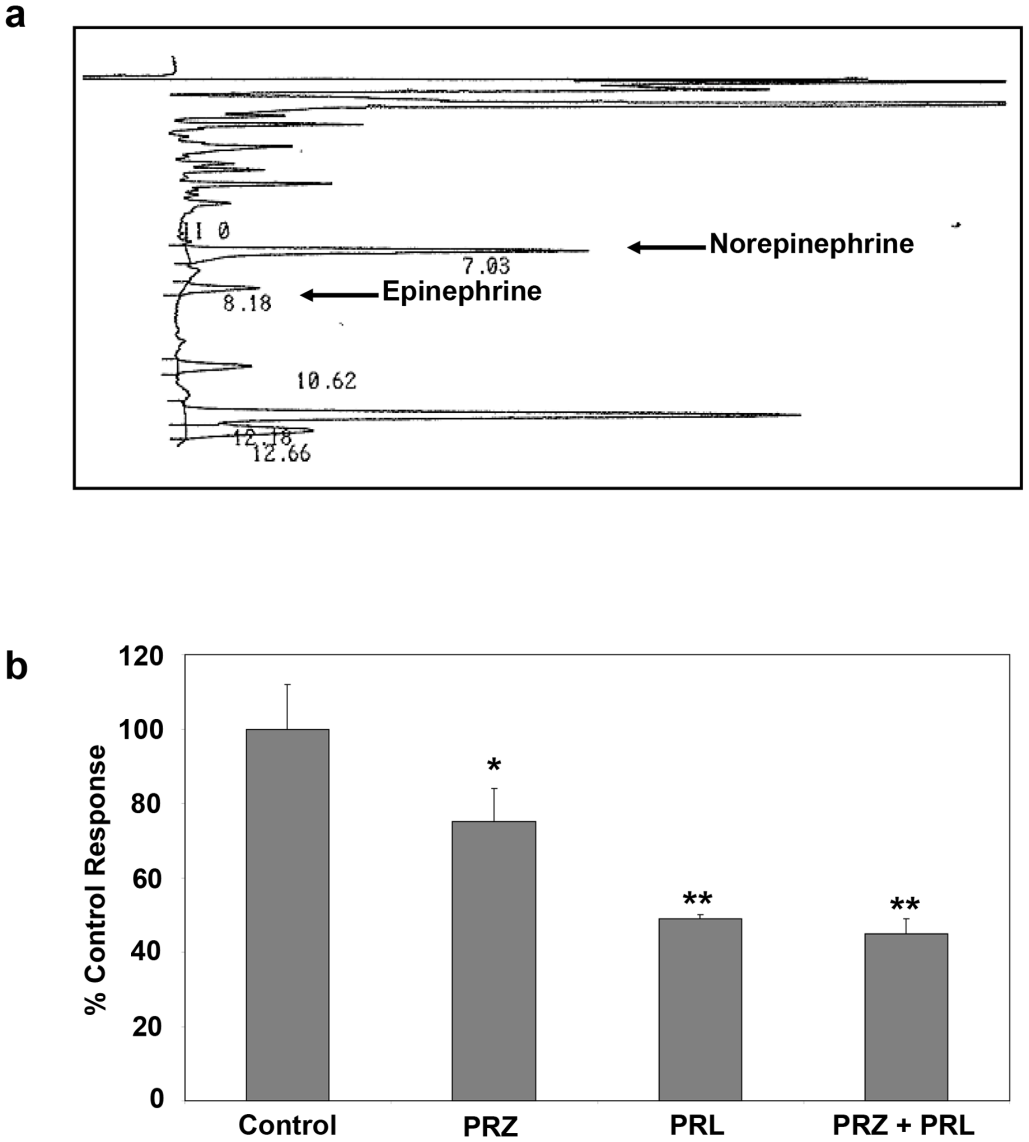
Primary hHSC express the cathecholamine synthesising enzyme dopamine-β-hydroxylase and synthesize/release NE plus EPI to regulate hHSC basal growth in culture. **a**) We first established by immunohistochemistry that hHSC expressed dopamine-β-hydroxylase (DBH) (*data not shown*). HPLC analysis of hHSC conditioned medium showed hHSC release of NE and EPI with NE more abundantly produced than EPI. **b**) Activated hHSC cultured basally for 48hours with PRZ (10 μM) or PRL (10 μM) singly or in combination significantly had reduced basal proliferation. Proliferation under each treatment condition was normalised to the corresponding serum free control in each experiment; *p<0.05, **p<0.0001, each bar represents mean ±SEM, n = 3.

Although murine HSC synthesize and release NE in culture and use this endogenously released NE for optimal basal growth [2], the existence of a similar pathway in hHSC is not known. We therefore examined by immunohistochemistry the expression of DBH, the rate limiting enzyme in the biosynthesis of cathecholamines. DBH was expressed by near normal livers and markedly upregulated in livers with NASH cirrhosis compared to near normal controls (mean counts of DBH positive cells 17±1 vs. 2±2, n = 5, p<0.001). HPLC analysis of hHSC conditioned medium showed that hHSC release NE and EPI, with NE released at about 83±9 pg/ml, more abundantly produced than EPI (Figure 5a). To investigate the importance of endogenous NE and EPI and the functionality of hHSC-expressed adrenoceptors, we cultured hHSC with PRZ, a predominant α1-adrenoreceptor antagonist, and PRL a predominant β-adrenoceptor antagonist. PRZ (10 μM) and PRL (10 μM) singly or combined significantly reduced hHSC basal growth (Figure 5b). Therefore, hHSC endogenously produced cathecholamines are important for hHSC basal growth via hHSC expressed adrenoceptors.

### Exogenous NE, EPI and NPY stimulate proliferation of hHSC through p38 MAP, PI3K and MEK

**Figure 6:**
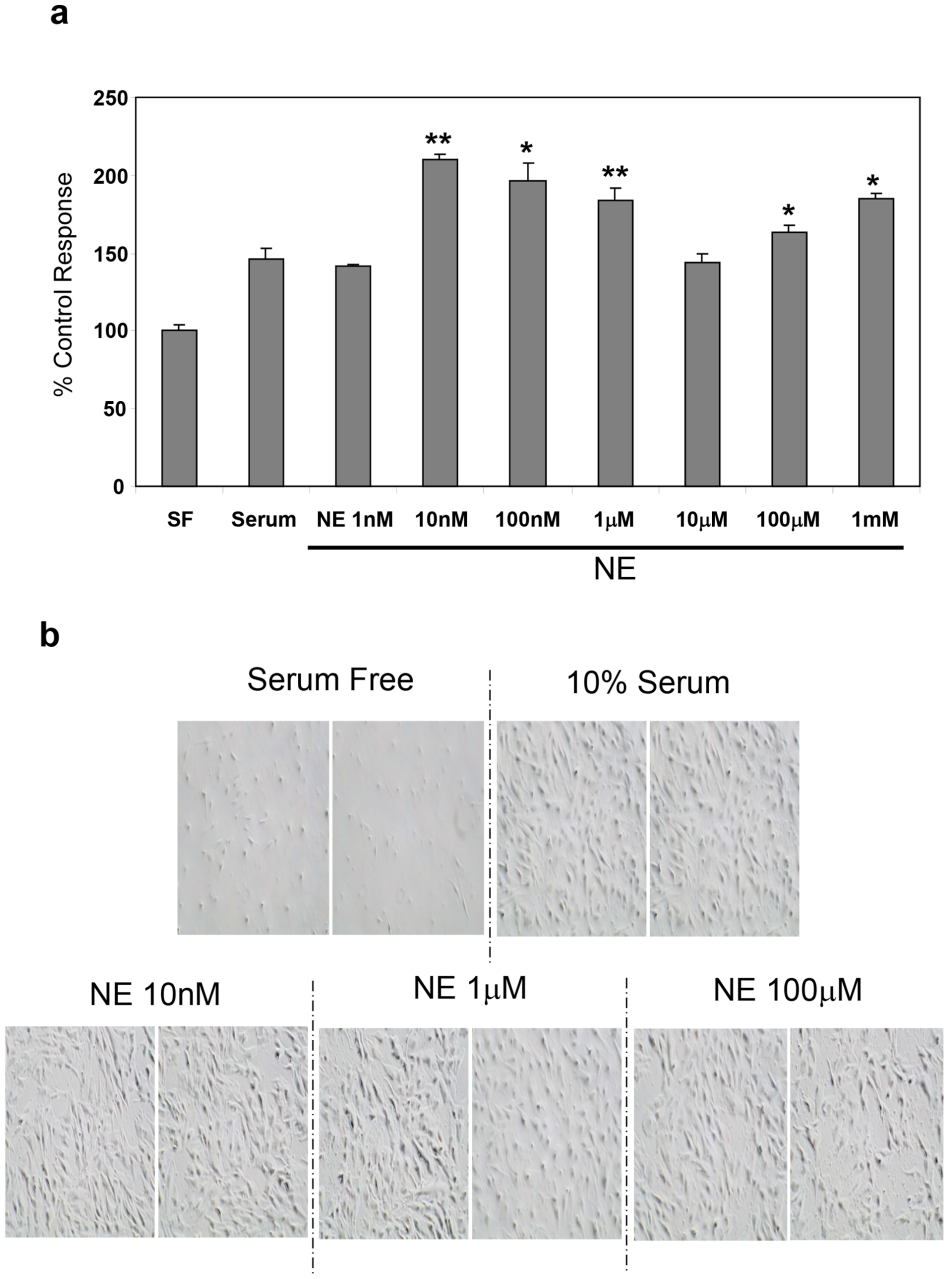
Exogenous NE stimulates proliferation of hHSC. **a**) NE induced a dose-dependent, biphasic, enhancement of proliferation of hHSC, with a maximal effect at 10 nM. Each bar represents mean ± SEM of triplicate responses in one typical experiment, expressed as a percentage of the response in serum free control wells. Similar results have been obtained on at least three other occasions; SF, serum free; * p<0.001, ** p<0.0001. **b**) Phase contrast micrographs are shown as visual confirmation of actual cell number increase in the presence of increasing concentrations of NE.

**Figure 7:**
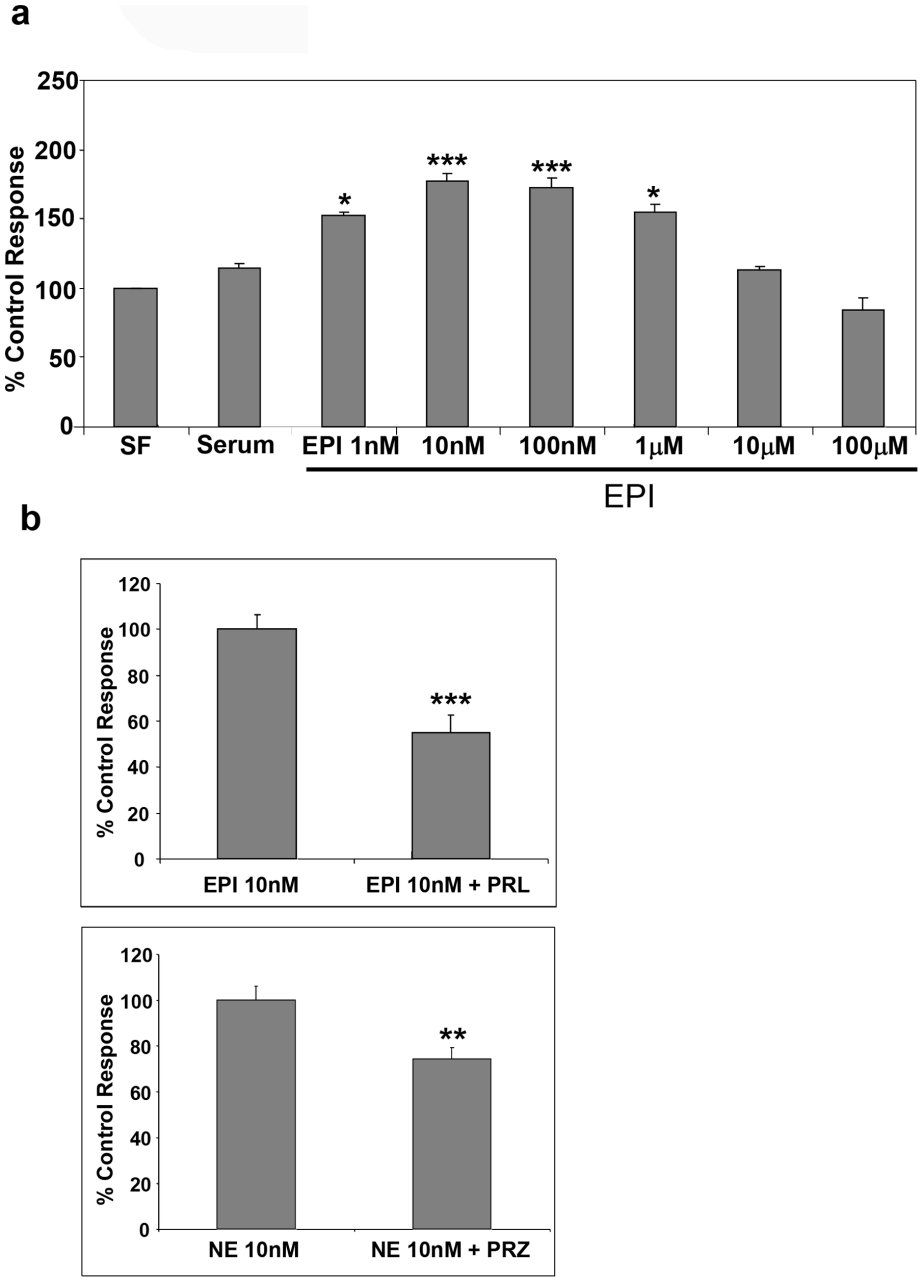
Exogenous EPI stimulate proliferation of hHSC. **a**) EPI, as with NE, similarly induced proliferation of cultured activated hHSC, at EPI concentrations of 1 nM–1 µM with a maximal effect at 10 nM. **b**) Prazosin (10 µM) significantly inhibited NE induced hHSC proliferation. PRL (10 µM) also inhibited EPI induced hHSC proliferation. Results are expressed as a percentage of each agonist control (100%).

**Figure 8:**
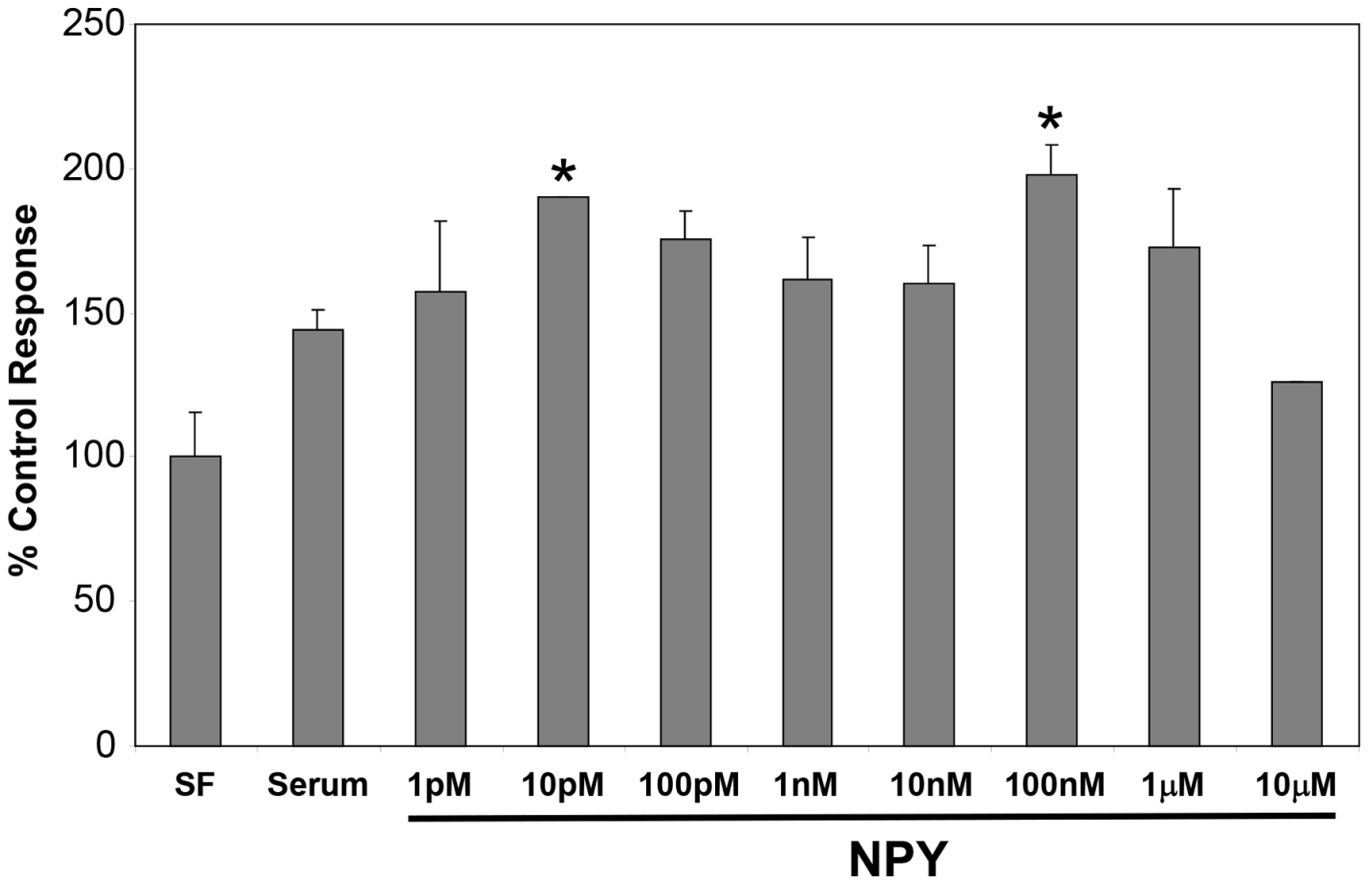
Exogenous NPY stimulate proliferation of hHSC. NPY induced proliferation of cultured activated hHSC, at concentrations as low as 10 pM. Results are expressed as a percentage of each agonist control (100%).

We then investigated whether exogenous NE and EPI as with murine HSC [2] induced hHSC proliferation. Activated hHSC were cultured for 48 hours in serum free medium, as negative controls, or in medium with 10% FBS as controls or with 10% FBS plus varying concentrations of NE (1 nM–1 mM). NE induced a dose-dependent, biphasic, enhancement of proliferation of hHSC with maximal effect at 10 nM (Figure 6a, b). EPI similarly induced hHSC proliferation at 1 nM – 1 mM with maximal effect at 10 nM (Figure 7a). The effect of EPI on hHSC proliferation was mostly mediated by β-adrenoceptors, since PRL significantly inhibited it (Figure 7b, top panel) whilst the effect of NE on hHSC proliferation was mostly mediated by α-adrenoceptors, since PRZ significantly inhibited NE induced proliferation (Figure 7b, bottom panel). NPY as with NE and EPI also induced biphasic hHSC proliferation with concentrations as low as 10 pM inducing significant increases in hHSC proliferation (Figure 8).

**Figure 9:**
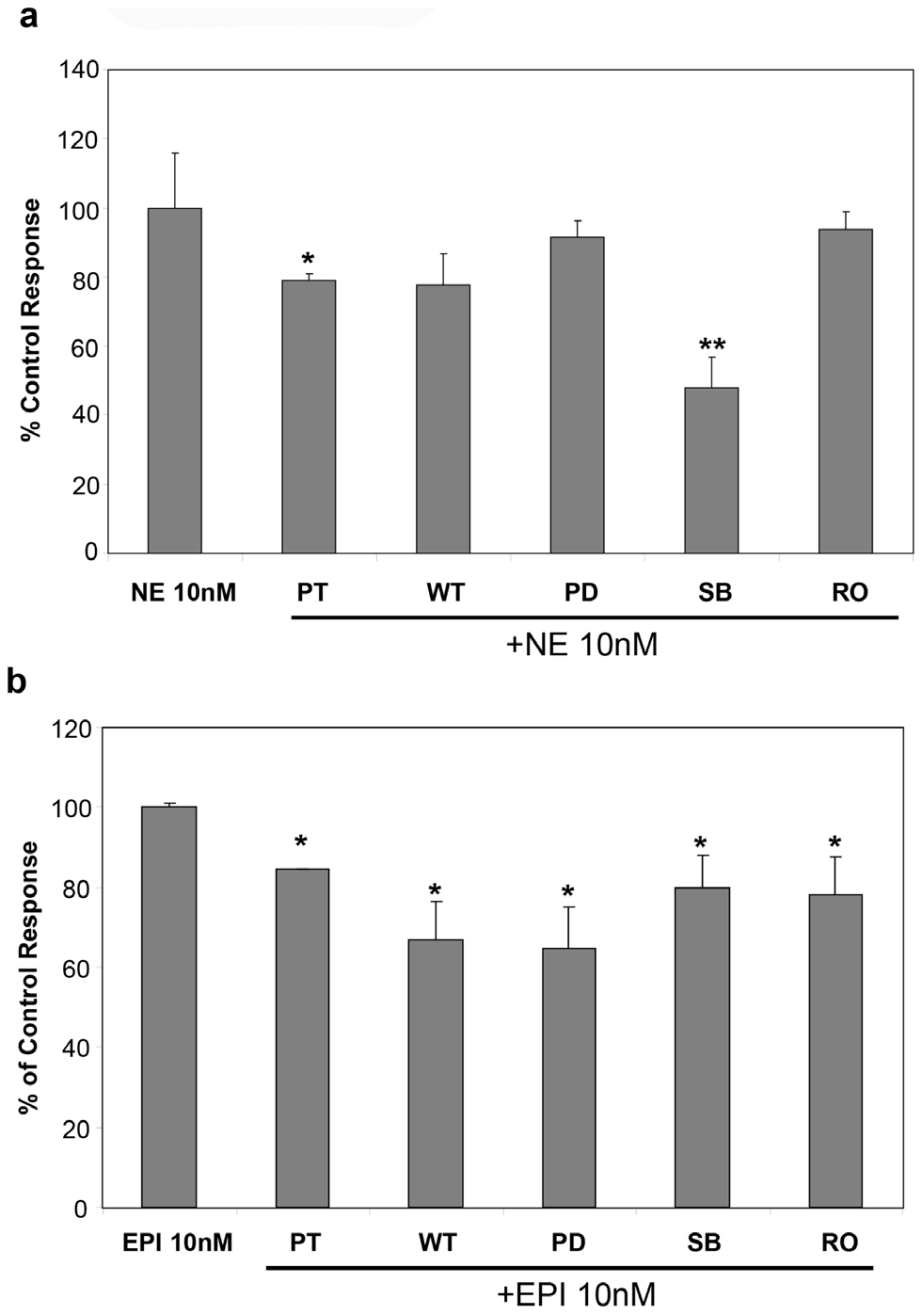
Exogenous NE and EPI stimulate proliferation of hHSC through p38 MAP, PI3K and MEK. **a**) Pre-treatment for 2 hours with PT (G-protein inhibitor, 100 nM) and SB203580 (p38MAP inhibitor, 10 µM) significantly reduced NE induced hHSC proliferation, with WT wortmannin (PI3K inhibitor, 100 nM), and PD98059 (MEK inhibitor, 100 nM) also slightly but non-significantly reducing NE-induced proliferation. **b**) The proliferative effects of EPI were also inhibited by PT and furthermore by WT, PD and RO (PKC inhibitor, 1 µM).

**Figure 10:**
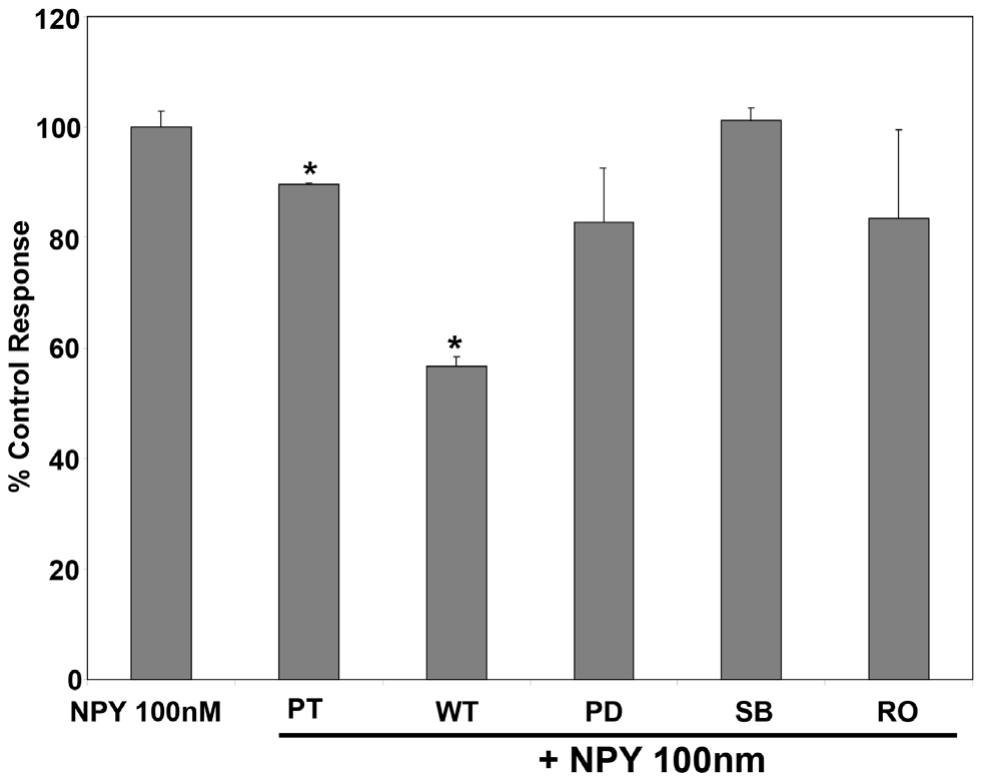
Exogenous NPY stimulate proliferation of hHSC through p38 MAP, PI3K and MEK. NPY proliferative effects on hHSC were inhibited by PT and WT. Results (mean ±SEM of triplicate responses in one typical experiment) are expressed as a percentage of the response in serum free control wells. Similar results have been obtained on at least three other occasions. SF, serum free; *p<0.05, **p<0.01, ***p<0.001.

To investigate the role of the G-protein, PI3K, MEK, p38 MAPK and PKC intra-cellular signalling pathways on NE and EPI-induced proliferation of hHSC, we now examined the effect of their respective inhibitors at the concentrations shown: pertussis toxin (PT, 100 ng/ml); wortmannin (WT, 100 nM); PD98059 (PD, 20nM); SB202190 (SB, 10 μM) and RO-320432 (RO, 1 μM) as previously detailed [2], [17]. Pertussis toxin pre-treatment significantly reduced NE induced hHSC proliferation, while pre-treatment with wortmannin, PD98059 and RO also slightly but non-significantly reduced NE-induced proliferation. However, pre-treatment with SB202190 markedly and significantly inhibited NE-induced hHSC proliferation (Figure 9a). Therefore, the proliferative effects of the predominant α1-adrenoceptor agonist NE, on hHSC, are mediated by G-protein coupled adrenoceptors with downstream effects mostly involving p38 MAPK. Conversely, the proliferative effects of the predominant β-agonist EPI involves G-protein coupled adrenoceptors mediated downstream by PI-3K and MEK with minor contributions from p38 MAP and PKC, since EPI effects were inhibited by PT and furthermore by WT and PD (Figure 9b). The hHSC NPY receptors, as with the hHSC adrenoceptors, appear also to be coupled to G-proteins with downstream pathways involving PI-3K since PT and WT inhibited NPY induced hHSC proliferation (Figure 10).

### Lack of Endogenous NE increases apoptosis of hHSC

**Figure 11:**
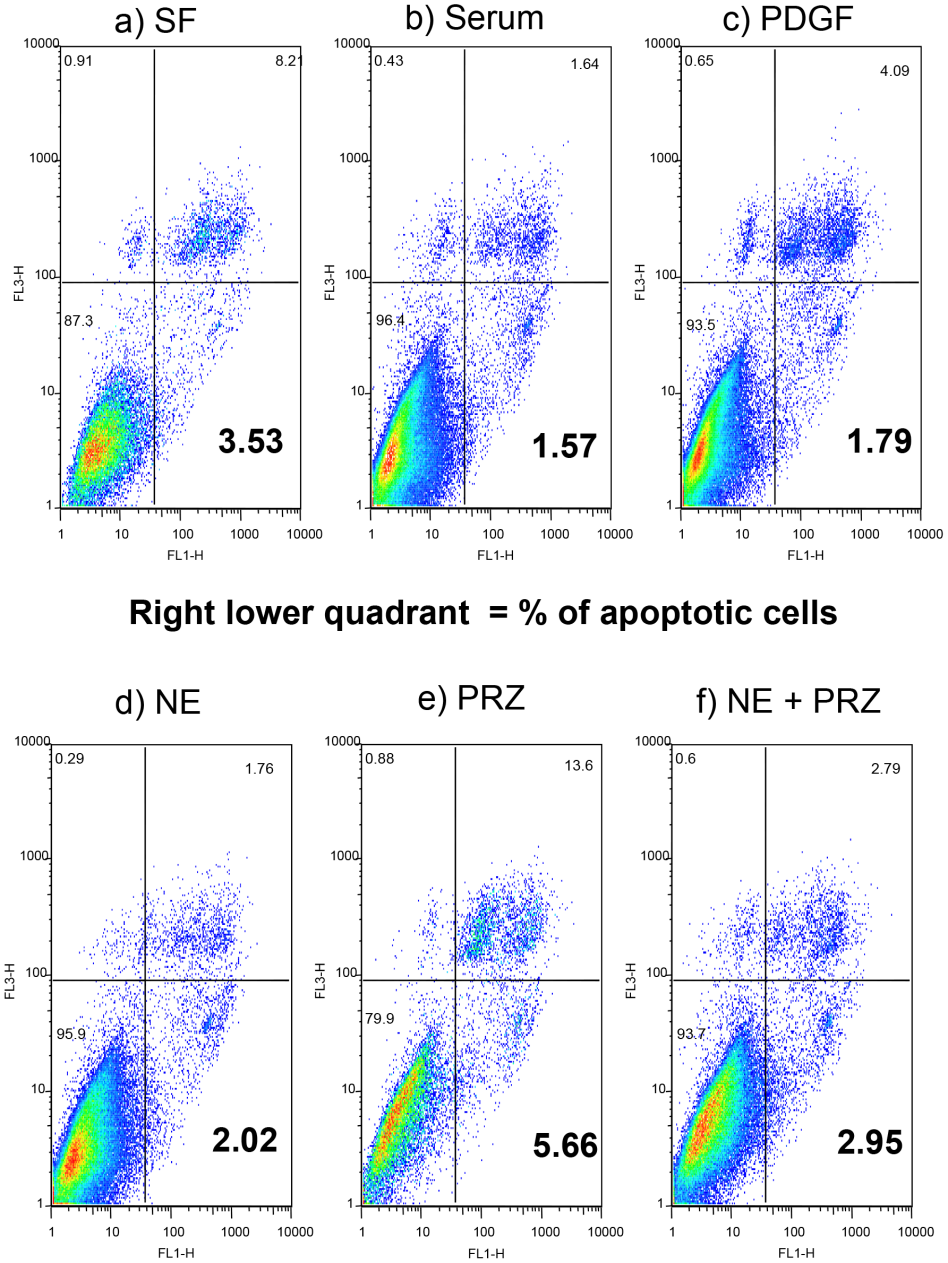
Lack of Endogenous NE increases apoptosis of hHSC. Absence of serum induced marked apoptosis (**a**), reduced by presence of serum (**b**), PDGF (**c**) or NE (**d**). PRZ markedly induced hHSC apoptosis more than serum deprivation (**e**), the effect of PRZ was abrogated by NE (**f**). Results shown are from 1 typical experiment. Similar results have been obtained on at least 2 other occasions. PRZ significantly induced apoptosis compared to serum alone or compared to NE alone (Serum alone 3±0.6% vs PRZ 11±3%, p<0.05,n = 3; NE 4±0.2% vs PRZ 11±3%, p<0.05,n = 3).

In the absence of serum (serum free, SF) (Figure 11a) as expected, marked apoptosis was observed, which was reduced by the presence of serum (Figure 11b), platelet derived growth factor, PDGF (Figure 11c) or NE (Figure 11d). PRZ markedly induced hHSC apoptosis to an even a greater extent than serum deprivation (Figure 11e). The effect of PRZ was abrogated by NE (Figure 11f). Therefore, hHSC endogenously produced NE and exogenously applied NE appears to be a survival factor for hHSC.

### Exogenous NE induced collagen expression through TGF-β

**Figure 12:**
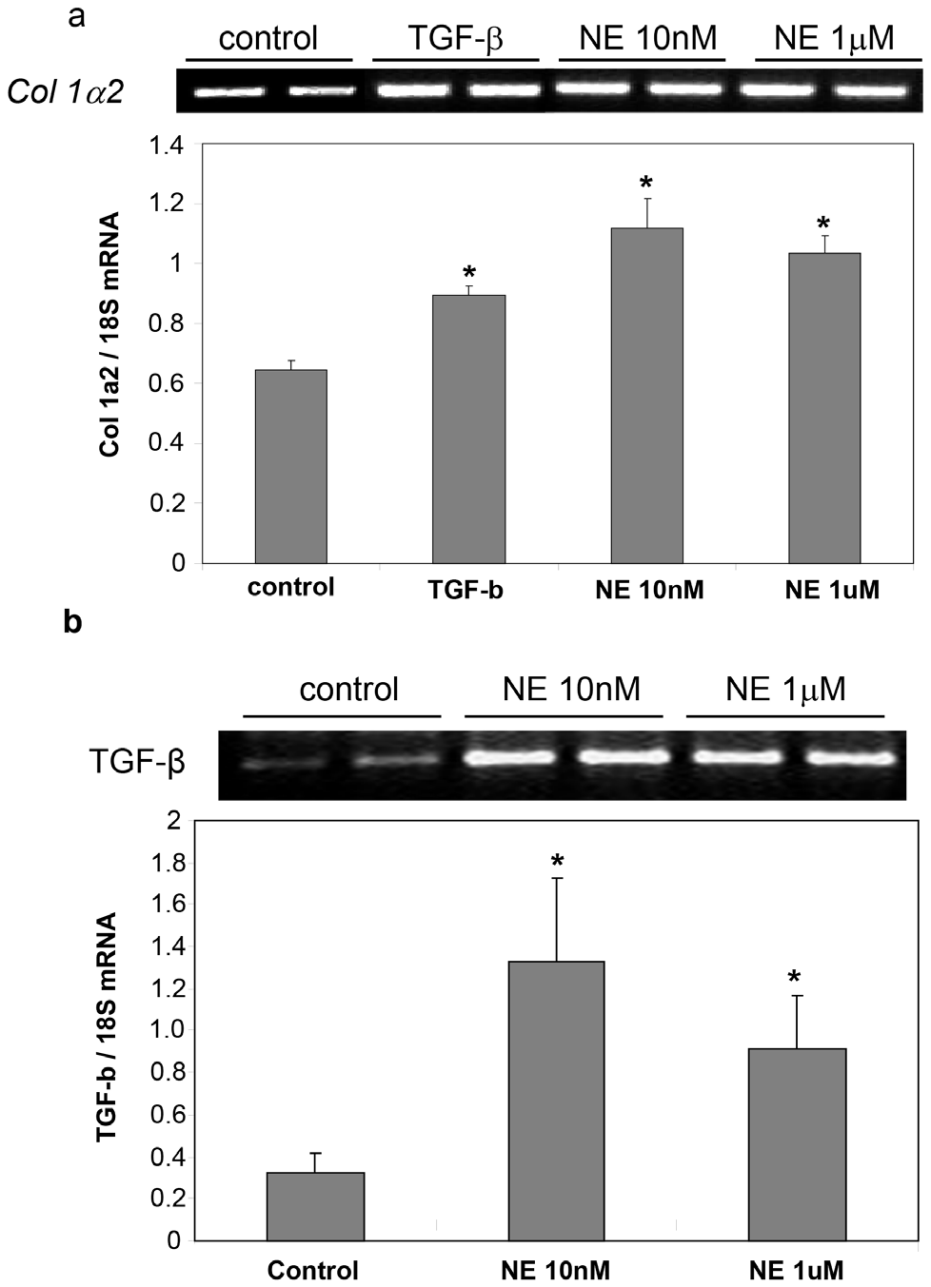
Exogenous NE induced collagen expression through TGF-β. **a**) NE upwards of 10nM significantly increased hHSC *collagen-1a2* gene expression, with absence of serum and TGF-β as negative and positive controls. **b**) NE significantly induced expression *TGF-β.* Results (mean ±SEM of triplicate responses in one typical experiment) are expressed as a percentage of the response in serum free control wells, vs SF, *p<0.05. Similar results have been obtained on at least three other occasions.

We now investigated whether NE applied to hHSC upregulated collagen gene expression in tandem with increased proliferation. NE significantly increased collagen-1α2 gene expression, at concentrations upwards of 10 nM (Figure 12a). Additionally, NE induced expression of TGF-β (Figure 12b), indicating that at least some of the increased collagen expression could be mediated through TGF-β.

### Pro-fibrotic effects of NE on hHSC are not mediated through leptin, IL-4 or IL-13 and NE does not induce production of IL-10

Besides TGF-β, leptin, IL-4 and IL-13 [25], [26] are also involved in hepatic fibrogenesis consequent to various aetiologies and IL-10 may be anti-fibrotic [25]–[28]. To determine if leptin, IL-4 or IL-13 are upregulated by NE in order to explain the pro-fibrotic effect of NE, and whether there is simultaneous induction of IL-10 by NE, we initially assayed by quantitative RT-PCR the expression of these factors by hHSC cultured under conditions identical to those used above to detect NE effects on hHSC proliferation. We found no appreciable evidence of induction by NE of leptin, IL-4, IL-10 or IL-13 gene expression (results not shown). We also confirmed by ELISA that there was no appreciable induction of leptin or IL-13 protein (results not shown). Therefore, the pro-fibrotic effect of NE on hHSC does not involve appear to involve leptin, IL-4 or IL-13. These results are in keeping with our previous findings that in HSC from mice NE induced pro-fibrogenic without a requirement for leptin [4].

## Discussion

We have shown here that hHSC express adrenoceptors, which are markedly upregulated in human livers with NAFLD cirrhosis. Activated hHSC in culture express α1A, β1, β2 and β3 adrenoceptors, while no convincing expression of α1B or α1D adrenoceptors was observed. This is to our knowledge the first comprehensive report of the adrenoceptor profile of primary hHSC including their expression of β3-adrenoceptors. hHSC also robustly express Y1, Y4, and Y6 NPY receptors with little expression of Y2 and Y5. These receptors are functional and are possibly implicated in NAFLD fibrosis, since patients with F0 fibrosis had reduced expression of α1A, β1, β2 and β3 adrenoceptors or Y1, Y4 and Y6 NPY receptors, whereas patients with NAFLD F4 fibrosis (cirrhosis) had exuberant expression of α1A, β1, β2 and β3 adrenoceptors plus Y1, Y4 and Y6 NPY receptors. These results suggest also that hHSC are potential targets for the pathogenic effect of catecholamines in the liver, consistent with recent data indicating that catecholamines contribute to tissue repair and inflammation, in addition to regulating vascular homeostasis [29]. Furthermore, hHSC express functional dopamine-β-hydroxylase and synthesize/release the cathecholamines NE plus EPI to regulate hHSC basal growth since PRZ (α1-adrenoreceptor antagonist) and PRL (β-adrenoceptor antagonist) reduced hHSC basal growth. Endogenous NE is also necessary for hHSC survival since there is markedly increased hHSC apoptosis in the presence of PRZ. DBH expression was markedly upregulated in livers with NAFLD cirrhosis.

NE is known to stimulate key intracellular kinases and signalling pathways implicated in the hepatic fibrogenesis [add Ref 2, [3]. Here we found that exogenous NE, EPI and NPY also regulate hHSC fibrogenic functions since NE, EPI and NPY dose-dependently induced hHSC proliferation through G-protein coupled receptors. The downstream effects of NE on hHSC are shown here to be mediated by p38 MAP and that of EPI mediated most likely by PI3K and MEK, with perhaps some minor contribution from p38 MAP and PKC. The involvement of these latter 2 pathways in hHSC proliferation needs clarifying in future experiments. The intra-cellular pathways involved in the actions of NE and EPI on hHSC as shown here, are similar to those reported for their actions in murine HSC and other cell types [2], [30]. Importantly, for our finding that the intra-cellular action of EPI on hHSC probably involves MEK; the MEK inhibitor PD98059 has recently been shown to reduce injury and fibrosis in a murine model of lung fibrosis [31]. Additionally, the proliferative effects of NPY on hHSC as shown here are similar to previous findings of the effect of NPY on murine HSC [5], but the current findings are to our knowledge the first comprehensive description of NPY receptors on HSC and the signalling pathways therein.

NE and EPI induced hHSC proliferation were maximal at 10nM. NE induced hHSC proliferation is an α-adrenoceptor mediated (PRZ inhibited) whilst EPI induced hHSC proliferation is β-adrenoceptor mediated (PRL inhibited). NPY as with NE and EPI dose-dependently induced hHSC proliferation with concentrations as low as 10 pM inducing significant increases in hHSC proliferation. Exogenously administered NE and EPI, moreover, induced collagen expression through TGF-β because NE and EPI applied to hHSC upregulated collagen gene expression in tandem with increased proliferation. Therefore, at least some of the increased collagen expression induced by NE and EPI in hHSC is mediated through TGF-β, in line with our previous study in mice [2]. It has been shown previously that HSC secrete pro-inflammatory chemokines such as interleukins [32], [33] and inflammatory mediators derived from lymphocytes can stimulate HSC fibrogenic potential [33], such that chemokine secretion by NE might enhance inflammation in chronic liver disease. In our study however, we did not find appreciable evidence of induction by NE of leptin, IL-4, IL-10 or IL-13. In support of our findings here, a study using a Mdr2(−/−) mouse model, displaying blockade of *β*-adrenoceptors has been shown to be a promising therapeutic strategy in the treatment of human primary sclerosing cholangitis [34]. Moreover, *β*-adrenoceptors mediate liver NE modulation of pro-inflammatory responses, cardiac dysfunction and portal hypertension in cirrhotic patients and rodent models of the disease [35]–[39]. In summary, our study clearly shows that hHSC express adrenergic and NPY receptors which are upregulated *in vivo* with fibrosis. Additionally, hHSC synthesise and require the cathecholamines NE and EPI for optimal survival and NE, EPI and NPY stimulate the proliferation of hHSC with NE also shown to induce collagen gene expression in cultured hHSC. These findings suggest therefore that adrenergic and NPY antagonists may be novel anti-fibrotic agents in NAFLD.

## References

[1] Lee Y, Friedman SL (2010) Fibrosis in the liver: acute protection and chronic disease. Prog Mol Biol Transl Sci 97: 151–200. 21074733 10.1016/B978-0-12-385233-5.00006-4

[2] Oben JA, Roskams T, Yang S, Lin H, Sinelli N, et al (2004) Hepatic fibrogenesis requires sympathetic neurotransmitters. Gut 53: 438–445. 14960531 10.1136/gut.2003.026658 PMC1773985

[3] Sancho-Bru P, Bataller R, Colmenero J, Gasull X, Moreno M, et al (2006) Norepinephrine induces calcium spikes and proinflammatory actions in human hepatic stellate cells. Am J Physiol Gastrointest Liver Physiol 291: G877–884. 16782692 10.1152/ajpgi.00537.2005

[4] Oben JA, Roskams T, Yang S, Lin H, Sinelli N, et al (2003) Norepinephrine induces hepatic fibrogenesis in leptin deficient ob/ob mice. Biochem Biophys Res Commun 308: 284–292. 12901866 10.1016/s0006-291x(03)01360-3

[5] Oben JA, Yang S, Lin H, Ono M, Diehl AM (2003) Norepinephrine and neuropeptide Y promote proliferation and collagen gene expression of hepatic myofibroblastic stellate cells. Biochem Biophys Res Commun 302: 685–690. 12646223 10.1016/s0006-291x(03)00232-8

[6] Lundberg JM, Torssell L, Sollevi A, Pernow J, Theodorsson Norheim E, et al (1985) Neuropeptide Y and sympathetic vascular control in man. Regul Pept 13: 41–52. 3841402 10.1016/0167-0115(85)90085-0

[7] Cavadas C, Cefai D, Rosmaninho-Salgado J, Vieira-Coelho MA, Moura E, et al (2006) Deletion of the neuropeptide Y (NPY) Y1 receptor gene reveals a regulatory role of NPY on catecholamine synthesis and secretion. Proc Natl Acad Sci U S A 103: 10497–10502. 16798884 10.1073/pnas.0600913103 PMC1502486

[8] Young JB, Landsberg L (1977) Suppression of sympathetic nervous system during fasting. Science 196: 1473–1475. 867049 10.1126/science.867049

[9] Hall JE, da Silva AA, do Carmo JM, Dubinion J, Hamza S, et al (2010) Obesity-induced hypertension: role of sympathetic nervous system, leptin, and melanocortins. J Biol Chem 285: 17271–17276. 20348094 10.1074/jbc.R110.113175 PMC2878489

[10] Rumantir MS, Vaz M, Jennings GL, Collier G, Kaye DM, et al (1999) Neural mechanisms in human obesity-related hypertension. J Hypertens 17: 1125–1133. 10466468 10.1097/00004872-199917080-00012

[11] Burt AD, Tiniakos D, MacSween RN, Griffiths MR, Wisse E, et al (1989) Localization of adrenergic and neuropeptide tyrosine-containing nerves in the mammalian liver. Hepatology 9: 839–845. 2565863 10.1002/hep.1840090608

[12] Anstee QM, Targher G, Day CP (2013) Progression of NAFLD to diabetes mellitus, cardiovascular disease or cirrhosis. Nat Rev Gastroenterol Hepatol. 10.1038/nrgastro.2013.41 23507799

[13] James O, Day C (1999) Non-alcoholic steatohepatitis: another disease of affluence. Lancet 353: 1634–1636. 10335777 10.1016/S0140-6736(99)00163-4

[14] Charlton M (2004) Nonalcoholic fatty liver disease: a review of current understanding and future impact. Clin Gastroenterol Hepatol 2: 1048–1058. 15625647 10.1016/s1542-3565(04)00440-9

[15] Oben J, Nikolopoulos A, Paulon E (2008 ) Non Alcoholic Fatty Liver Disease CPD Bulletin - Biochemistry. 9: 47–53.

[16] Rodriguez B, Torres DM, Harrison SA (2012) Physical activity: an essential component of lifestyle modification in NAFLD. Nat Rev Gastroenterol Hepatol 9: 726–731. 23090329 10.1038/nrgastro.2012.200

[17] Soeda J, Morgan M, McKee C, Mouralidarane A, Lin C, et al (2012) Nicotine induces fibrogenic changes in human liver via nicotinic acetylcholine receptors expressed on hepatic stellate cells. Biochem Biophys Res Commun 417: 17–22. 22108052 10.1016/j.bbrc.2011.10.151

[18] Oben JA, Yang S, Lin H, Ono M, Diehl AM (2003) Acetylcholine promotes the proliferation and collagen gene expression of myofibroblastic hepatic stellate cells. Biochem Biophys Res Commun 300: 172–177. 12480538 10.1016/s0006-291x(02)02773-0

[19] Brunt EM, Kleiner DE, Wilson LA, Belt P, Neuschwander-Tetri BA, et al (2011) Nonalcoholic fatty liver disease (NAFLD) activity score and the histopathologic diagnosis in NAFLD: distinct clinicopathologic meanings. Hepatology 53: 810–820. 21319198 10.1002/hep.24127 PMC3079483

[20] Pazienza V, Vinciguerra M, Andriulli A, Mangia A (2010) Hepatitis C virus core protein genotype 3a increases SOCS-7 expression through PPAR-{gamma} in Huh-7 cells. J Gen Virol 91: 1678–1686. 20357037 10.1099/vir.0.020644-0

[21] Deblon N, Bourgoin L, Veyrat-Durebex C, Peyrou M, Vinciguerra M, et al (2012) Chronic mTOR inhibition by rapamycin induces muscle insulin resistance despite weight loss in rats. Br J Pharmacol 165: 2325–2340. 22014210 10.1111/j.1476-5381.2011.01716.x PMC3413866

[22] Benegiamo G, Mazzoccoli G, Cappello F, Rappa F, Scibetta N, et al (2013) Mutual Antagonism between Circadian Protein Period 2 and Hepatitis C Virus Replication in Hepatocytes. PLoS One 8: e60527. 23593233 10.1371/journal.pone.0060527 PMC3620463

[23] Rappa F, Greco A, Podrini C, Cappello F, Foti M, et al (2013) Immunopositivity for histone macroH2A1 isoforms marks steatosis-associated hepatocellular carcinoma. PLoS One 8: e54458. 23372727 10.1371/journal.pone.0054458 PMC3553099

[24] Cassiman D, van Pelt J, De Vos R, Van Lommel F, Desmet V, et al (1999) Synaptophysin: A novel marker for human and rat hepatic stellate cells. Am J Pathol 155: 1831–1839. 10595912 10.1016/S0002-9440(10)65501-0 PMC1866940

[25] Aoudjehane L, Pissaia A Jr, Scatton O, Podevin P, Massault PP, et al (2008) Interleukin-4 induces the activation and collagen production of cultured human intrahepatic fibroblasts via the STAT-6 pathway. Lab Invest 88: 973–985. 18626468 10.1038/labinvest.2008.61

[26] Saxena NK, Yang Y, Floyd J, Anania F (2002) Leptin is mitogenic , anti-apoptotic and increases fibrogenic response genes in rat hepatic stellate cells. Hepatology 36 36: 316A.

[27] Zhang LJ, Zheng WD, Chen YX, Huang YH, Chen ZX, et al (2007) Antifibrotic effects of interleukin-10 on experimental hepatic fibrosis. Hepatogastroenterology 54: 2092–2098. 18251166

[28] Borthwick LA, Wynn TA, Fisher AJ (2013) Cytokine mediated tissue fibrosis. Biochim Biophys Acta 1832: 1049–1060. 23046809 10.1016/j.bbadis.2012.09.014 PMC3787896

[29] Bonnefont-Rousselot D, Mahmoudi A, Mougenot N, Varoquaux O, Le Nahour G, et al (2002) Catecholamine effects on cardiac remodelling, oxidative stress and fibrosis in experimental heart failure. Redox Rep 7: 145–151. 12189044 10.1179/135100002125000389

[30] Sivamani RK, Lam ST, Isseroff RR (2007) Beta adrenergic receptors in keratinocytes. Dermatol Clin 25: 643–653, x. 10.1016/j.det.2007.06.012 PMC2169297 17903623

[31] Galuppo M, Esposito E, Mazzon E, Di Paola R, Paterniti I, et al (2011) MEK inhibition suppresses the development of lung fibrosis in the bleomycin model. Naunyn Schmiedebergs Arch Pharmacol 384: 21–37. 21533992 10.1007/s00210-011-0637-7

[32] Marra F (1999) Hepatic stellate cells and the regulation of liver inflammation. J Hepatol 31: 1120–1130. 10604588 10.1016/s0168-8278(99)80327-4

[33] Schwabe RF, Schnabl B, Kweon YO, Brenner DA (2001) CD40 activates NF-kappa B and c-Jun N-terminal kinase and enhances chemokine secretion on activated human hepatic stellate cells. J Immunol 166: 6812–6819. 11359840 10.4049/jimmunol.166.11.6812

[34] Strack I, Schulte S, Varnholt H, Schievenbusch S, Tox U, et al (2011) beta-Adrenoceptor blockade in sclerosing cholangitis of Mdr2 knockout mice: antifibrotic effects in a model of nonsinusoidal fibrosis. Lab Invest 91: 252–261. 20921947 10.1038/labinvest.2010.162

[35] Zapater P, Gomez-Hurtado I, Peiro G, Gonzalez-Navajas JM, Garcia I, et al (2012) Beta-adrenergic receptor 1 selective antagonism inhibits norepinephrine-mediated TNF-alpha downregulation in experimental liver cirrhosis. PLoS One 7: e43371. 22916250 10.1371/journal.pone.0043371 PMC3423372

[36] Vasina V, Giannone F, Domenicali M, Latorre R, Berzigotti A, et al (2012) Portal hypertension and liver cirrhosis in rats: effect of the beta3-adrenoceptor agonist SR58611A. Br J Pharmacol 167: 1137–1147. 22708587 10.1111/j.1476-5381.2012.02074.x PMC3492993

[37] Trebicka J, Hennenberg M, Schulze Probsting A, Laleman W, Klein S, et al (2009) Role of beta3-adrenoceptors for intrahepatic resistance and portal hypertension in liver cirrhosis. Hepatology 50: 1924–1935. 19842096 10.1002/hep.23222

[38] Theocharidou E, Krag A, Bendtsen F, Moller S, Burroughs AK (2012) Cardiac dysfunction in cirrhosis – does adrenal function play a role? A hypothesis. Liver Int 32: 1327–1332. 22292920 10.1111/j.1478-3231.2011.02751.x

[39] Moller S, Henriksen JH (2010) Cirrhotic cardiomyopathy. J Hepatol 53: 179–190. 20462649 10.1016/j.jhep.2010.02.023

